# Infective Endocarditis Due to an Organism Not Hitherto Described

**Published:** 1910-11-19

**Authors:** 


### Medicine.

### INFECTIVE ENDOCARDITIS DUE TO AN ORGANISM NOT HITHERTO DESCRIBED.

The bacteriology of infective or malignant endocarditis is complex. It is perfectly certain that the disease is not specific, but may be due to many different organisms. It is probable that the same microbe that is responsible for the acute simple endocarditis of ordinary rheumatic fever may sometimes be so virulent, or else the patient's resisting powers to it may be so poor that under certain circumstances, instead of producing a condition which rapidly recovers wholly or partially, it may lead to fungating endocarditis without there being any additional infection by any secondary micro-organisms. On the other hand, there is no doubt that many cases of infective endocarditis are due to secondary infection by such micro-organisms as streptococci, staphylococci, pneumococci, bacillus coli communis, and even diphtheria and typhoid bacilli. By reason of cultures made from blood withdrawn from a vein during life, the nature of the secondary organisms in these cases is becoming recognised with increasing frequency, and the importance of this lies not so much in respect to diagnosis, but in regard to treatment. If streptococci are found, benefit may be obtained by the use of anti-streptococcal serum; if other organisms are detected the proper vaccine has sometimes been employed with benefit in cases which presumably without the vaccine treatment would have died. Hence it is most important in all cases of the kind to determine the nature of the micro-organism present as early as possible.

### Dr. Pritchard's Case.

Dr. Pritchard has added to our knowledge of the bacteriology of infective endocarditis by showing that sometimes it may be due to bacteria which do not conform to any of the more generally recognised types. A paper published by him upon the subject will be found in the St. Bartholomew's Hospital Reports, vol. xlv. The clinical history of one case was as follows:—

T. G., a labourer, aged thirty-two, was admitted on January 27, 1908, complaining of weakness and of swelling of the legs. Previous to the onset of his present illness the patient had always enjoyed good health; he had never suffered from rheumatic fever. Approximately six weeks before his admission to the hospital he had complained of pain in the back, and many and various indefinite symptoms which he described as "influenza." The onset of his illness was apparently very gradual and ill-defined, for, although a man of good intelligence, he found difficulty in putting a date to it.

When first seen in hospital the patient had the appearance of one who had been for some time out of health though not acutely ill. He was pale, dyspnœic on exertion, and had lost flesh. His temperature was 99° F., his skin was moist, there was "pitting" over the shins, and some œdema of the ankles. The pulse was 92, of fair volume and diminished tension; the radial arteries were easily palpable. The area of cardiac dulness was increased, and this chiefly on the left side; the apex beat was situate in the fifth space, just outside the left nipple line. A systolic murmur of curious distribution was to be heard over the left side of the cardiac area; its maximum intensity was at a point 3½ inches from the sternum over the third rib on the left side; here it was harsh in quality and very loud. The murmur could also be heard at the apex, rapidly fading in intensity towards the axilla, and heard but very faintly at the angle of the left scapula. From time to time this murmur varied both in quality and in intensity, occasionally having a distinctly musical quality. It was thought to indicate a mural endocarditis; this was confirmed at the post-mortem examination. The second sound of the heart was well heard at the base, and was clear and well defined in the aortic area; there was no murmur audible during diastole.

The nervous, respiratory, and alimentary systems at the time of admission presented nothing worthy of note. The urine had a sp. gr. 1020; contained a cloud of albumen and a few granular casts. Throughout his illness fever was continuous, varying from 100° to 103° F., becoming subnormal just before death. The pulse varied from 92 to 140. The occurrence of embolism was a marked feature of the latter part of the illness. On one occasion the patient became suddenly aphasic and hemiplegic on the right side; later there were several attacks of hæmaturia with pain in the loins. At the end there was nothing eventful, death taking place from cardiac failure approximately eleven weeks from the onset of his illness.

At the post-mortem examination the usual lesions found in this disease were present. The heart weighed 17 oz.; the right side of the heart was much dilated, and the left ventricle showed marked hypertrophy. On opening the left auricle two large papillomatous masses of vegetations were seen spreading right up into the cavity, one arising from each half of the mitral valve. A large patch of mural endocarditis was seen on the auricular wall opposite one of these masses. The mitral valve was evidently the seat of old endocarditis, the cusps being thick and fused together. The chordæ tendineæ were short and thick, and encrusted over with vegetations. The aortic valves were normal. Signs of infarction were found in the brain, meninges, spleen, kidneys, and intestines. The kidneys showed the changes of a chronic parenchymatous nephritis.

On three occasions during life, blood was sent to the bacteriological laboratory for cultures to be made, and on each of these occasions an organism was obtained in pure culture. The organism proved to be a short, straight, slender, rod-shaped bacillus about 1.5 $\mu$ in length, of uniform thickness, occurring singly, and also tending to grow in chains. There was at first some question as to its motility. It was accordingly stained for flagella; these were not demonstrated. The staining process was then repeated, typhoid bacilli being used as a control on the same slide. Flagella were demonstrated in the case of the typhoid bacilli, but not in the case of this organism. Thus the bacillus must be regarded as non-motile.

The bacillus stained readily and uniformly with all the ordinary aniline dyes. It partly retained the stain in Gram's method.

The bacillus grew well at 37° C. There was at first no growth at the room temperature. Growth was obtained without access of oxygen, but this growth was not so marked as that which occurred in the presence of oxygen, the organism thus being a facultative anäerobe. The first subcultures from the original broth cultures were made on to blood-agar. The growth so obtained consisted of a number of small, round, discrete, but closely set, very transparent colonies about the size of those of the pneumococcus. After subcultivating for some days growth was obtained on ordinary agar, and after the organism had become accustomed to test-tube life scanty growth was obtained on gelatine in the cold. There was no liquefaction, and no gas formation in gelatine shake. The appearance of the colonies on all media was similar to that given above.

The organism showed no acid-forming properties and did not clot milk. It was put through the following sugar solutions: saccharose, lactose, raffinose, salicin, inulin, mannite, and glucose, with completely negative results. There was no change in neutral red. There was no evidence of spore formation. A rabbit inoculated intravenously fell ill and died upon the sixth day. Post-mortem no macroscopic changes were found. A bacillus identical with the one above described was obtained in films made from the peritoneal fluid and the spleen, and in cultures made from the heart's blood, peritoneal fluid, and spleen. A mouse inoculated subcutaneously died in twenty-four hours. Here also there were no macroscopic lesions, but cultures made from the heart's blood and the spleen yielded a similar organism. The organism was apparently non-pathogenic to the guinea-pig.

Cultures made from the heart's blood of the patient did not yield any growth; this was rather to be anticipated in view of the fact that the organism so easily died when exposed to cold in the laboratory. The patient had been dead 36 hours before the post-mortem was held. Sections made from the vegetations on the heart valves and appropriately stained showed them to contain the organism in great numbers. Every addition to our knowledge of the bacteriology of infective endocarditis is important, for it is a step towards the most likely method of curing the disease—namely, by the use of the proper vaccine or serum.

